# Radiation-induced osteosarcoma of the chest wall after treatment for unresectable thymoma

**DOI:** 10.1016/j.radcr.2023.07.076

**Published:** 2023-08-10

**Authors:** Siddharth Venkatraman, Edmund M. Weisberg, Elliot K. Fishman

**Affiliations:** a The Johns Hopkins University School of Medicine, 733 N Broadway, Baltimore, MD 21205, USA; b The Russell H. Morgan Department of Radiology and Radiological Science, Johns Hopkins University School of Medicine, Baltimore, MD, USA

**Keywords:** Radiation-induced osteosarcoma, Secondary osteosarcoma, Cinematic rendering, Computed Tomography, Thymoma

## Abstract

Secondary osteosarcoma is a rare complication of radiation therapy for a primary tumor. Here we report a unique presentation of radiation-induced osteosarcoma of the chest wall after radiation treatment for thymoma. This patient underwent multiple imaging studies, including magnetic resonance imaging and computed tomography with cinematic rendering. Diagnosis of osteosarcoma was confirmed through imaging features and histology. Several surgical procedures were performed to evaluate and attempt resection of the tumor, but ultimately the tumor location and involvement prevented adequate resection and chemotherapy was initiated. This case highlights the importance of identifying clear cumulative dose thresholds for radiation therapy and rare complications of radiotherapy.

## Introduction

Radiation therapy is among the primary curative modalities for several solid tumors. While rare, radiation-induced osteosarcoma is a potentially devastating iatrogenic complication of radiation therapy. Between 50% and 60% of radiation-induced sarcomas are osteosarcomas, and 0.01% to 0.03% of patients who receive radiation will develop an osteosarcoma [1]. These radiation-induced osteosarcomas are usually aggressive and associated with a worse prognosis compared with its sporadic counterpart due to the higher likelihood of a central tumor site, metastases, and incomplete surgical resection [2,3]. In this article, we describe a unique case of radiation-induced osteosarcoma of the chest wall presenting as mediastinal swelling and chest pain in a 51-year-old male treated with ionizing radiation for an unresectable thymoma. This case raises awareness of radiation-induced osteosarcoma and highlights the importance of the use of cinematic rendering to better identify and manage this rare complication.

## Case report

**Fig. 1:**
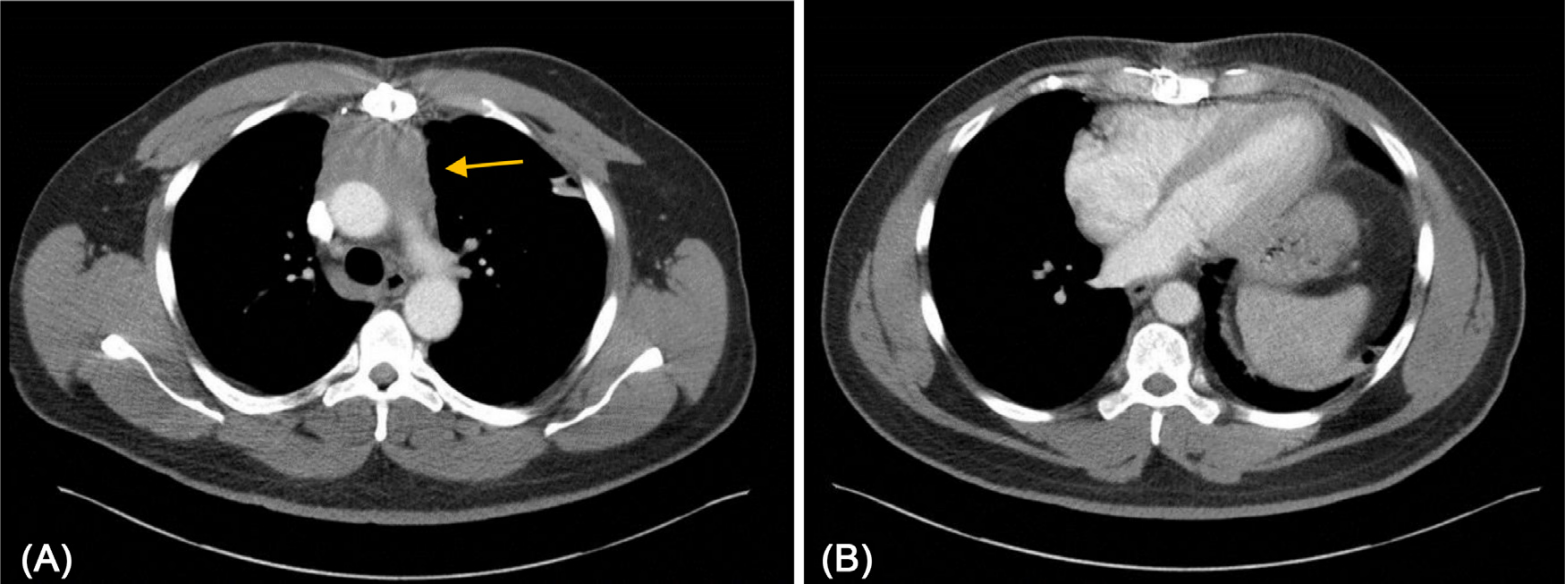
Axial CT imaging from 2016 showing (A) a homogeneous lobulated mass (yellow arrow) at the anterior mediastinum measuring 7.6 × 5.6 cm confirmed to be a thymoma. (B) normal presternal region prior to radiation therapy.

A 51-year-old male with a remote 2 pack-year smoking history and a prior diagnosis of thymoma presented with asymptomatic mediastinal swelling during a routine oncology surveillance appointment. His oncologic history included a stage III WHO grade AB thymoma, which presented as chest pain, dysphagia, visual changes, and muscle weakness suggestive of myasthenia gravis in 2016. Initial imaging in May 2016 revealed a 7.6 × 5.6 cm anterior mediastinal mass causing superior vena cava (SVC) syndrome (Figs. 1A and B). He underwent an elective mediastinal exploration and transsternal excisional biopsy in June 2016, but due to extensive involvement of the RV outflow tract, SVC, bilateral internal mammary arteries, and left phrenic nerve, the tumor was deemed unresectable. The patient received 4 cycles of cyclophosphamide (brand name: Cytoxan), doxorubicin (brand name: Adriamycin), and cisplatin (brand name: Platinol) combination chemotherapy from June to October 2016. He then underwent intensity-modulated pencil beam scanning proton therapy to the mediastinum in 1.8 Gy daily fractions for a total radiation dose of 68.4 Gy from January to March 2017. The thymoma was responsive, with a reduction of the tumor to 5.7 × 4.2 cm, and he remained in his usual state of health with regular follow-up over the next 5 years.

In May 2022, routine surveillance imaging showed an anterior chest wall subcutaneous soft tissue ossification, which was asymptomatic and thought to be myositis ossificans. At the time, the patient noted swelling of the anterior sternum but minimal pain from the mass. He denied any fevers, night sweats, cough, dysarthria, dyspnea, or hemoptysis. He has no known history of cancer in his family, and his past medical history is otherwise noncontributory. His physical examination was also otherwise unremarkable.

### Imaging studies

**Fig. 2:**
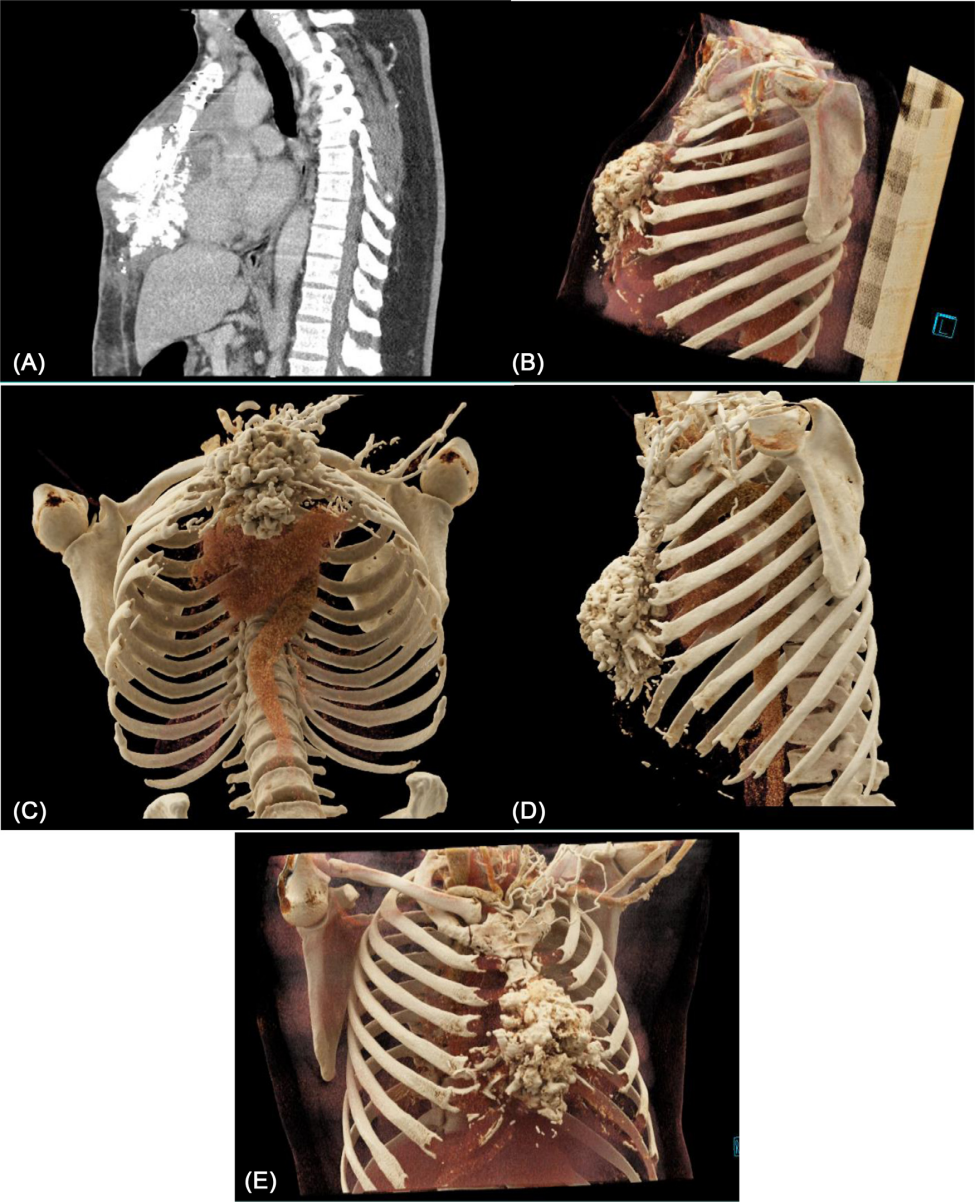
A) Sagittal CT scan demonstrates osteoid matrix involving chest wall, sternum, and retrosternal space, (B–E) Cinematic renderings define the extent of tumor matrix and involvement of sternum and chest wall.

A CT scan of the patient's chest in May 2022 demonstrated evidence of a rapidly evolving, heavily calcified, presternal mass measuring 7.2 × 10.5 × 12.8 cm (Fig. 2A). Cinematic rendering of the CT data further illustrated the size and extent of the mass (Figs. 2B–E). A PET/CT scan showed abnormal metabolic activity associated with the sternal mass suggesting a malignant periosteal reaction or heterotopic calcification. Histologic sections revealed a bone-forming neoplasm composed of epithelioid osteoblasts with moderate to severe cytologic atypia and scattered mitotic activity, confirming a high-grade secondary osteosarcoma arising from previously radiated bone. The patient completed 2 cycles of ifosfamide (brand name: Ifex) and etoposide (brand name: VePesid) chemotherapy from June 2022 to August 2022, followed by 2 cycles of cisplatin and doxorubicin chemotherapy ending in October 2022. A follow-up CT in November showed no change in the size of the osteosarcoma or the residual thymic mass. In December 2022, an operative resection of the osteosarcoma was attempted and subsequently aborted because the thymic and sternal masses remained adherent to the anterior heart. Six weeks after his operation, the patient was stable with an unchanged mediastinal mass and some minimal erythema and drainage from the incision site. He has since continued surveillance imaging of this thymoma, osteosarcoma, and calcified pulmonary nodules suspicious for metastatic spread.

In summary, this 51-year-old patient developed an unresectable secondary osteosarcoma of the chest wall due to his radiation treatment for an unresectable thymoma.

## Discussion

This is a rare case of radiation-induced osteosarcoma, occurring 5 years after chemoradiation for an unresectable thymoma. Osteosarcomas are 1 of the 3 major types of bone sarcomas, which also include chondrosarcomas and Ewing sarcoma. They can arise at any age, with adult cases occurring most often in the fifth or sixth decade of life. Radiation-induced sarcomas most often develop in the pelvis or the chest wall after a period of latency in contrast to primary osteosarcomas, which often arise in the long bones of the upper and lower extremities [4].

Radiotherapy can be considered a “double-edged sword.” Beyond its cytotoxic effects on malignant cells, radiation is a well-known mutagen. When used therapeutically for many inoperable thoracic tumors, lower doses at the periphery of the irradiation zone render these regions at the highest risk for malignancy because these cells undergo DNA damage without cell death [5]. A uniform mechanism for radiation-induced carcinogenesis remains poorly understood, and each tissue type has its own sensitivity to radiation [6]. Several studies have revealed that there is a threshold radiation dose required to induce bone sarcoma. Among 2383 radium-dial workers, all 64 bone sarcomas occurred in workers with more than 10 Gy of radiation [7]. An additional study of 40 radiation-induced osteosarcomas demonstrated that a median radiation dose of 50 Gy with a minimum dose of 20 Gy was required to induce malignant transformation [8]. Data remains limited on whether patients who develop radiation-induced osteosarcoma are genetically predisposed to be more susceptible to the effects of ionizing radiation. To date, research has shown an association between diseases of DNA repair and genome instability such as Costello syndrome or Nijmegen breakage syndrome, and the development of sarcomas, but these patients typically do not develop bone sarcomas [9].

The most common radiographic features seen in radiation-induced osteosarcoma are soft tissue extension of the tumor mass, lytic lesions of bone, and tumor osteoid matrix mineralization. However, radiographic features are not sufficient to differentiate between primary and secondary osteosarcoma; histologic findings and a thorough clinical and radiation therapy history should be obtained [10]. In 1999, Murray et al. revised the Cahan criteria for diagnosis of radiation-induced osteosarcoma to require that the sarcoma arises in the area included in the radiation field and the 5% isodose line, no evidence of sarcoma before radiation therapy, and confirmation that the sarcoma is histologically distinct from the original tumor [11].

In patients who present with radiation-induced osteosarcoma, treatment is usually with chemotherapy followed by a margin-negative surgical excision of the residual mass [4]. However, surgical dissection of nonextremity tumors can be difficult due to the fibrotic tissue in the irradiated area, larger tumor size, vascular and neurologic involvement, and reconstructive challenges [12]. These tumors also demonstrate a poor response to chemotherapy compared with their sporadic counterparts due to irradiation of the surrounding area. Even after surgery with curative intent, recurrence rates can be high. One study estimated that 31% will develop locoregional recurrence, while 28% can develop distant metastasis at a median follow-up time of 15 months [9]. Overall prognosis remains poor in patients with radiation-induced sarcomas, with a median survival of approximately 23 months [9].

One unique aspect of this case is the use of cinematic renderings of the patient's CT data as part of imaging evaluation and surgical planning. This case report is, to the best of our knowledge, one of the first uses of cinematic rendering for a radiation-induced osteosarcoma of the chest wall. Cinematic rendering incorporates a complex photorealistic lighting model and ray tracing to generate a 3D image with more accurate vascular mapping and estimation of lesion characteristics, such as depth and texture perception [13].

## Conclusion

Exposure to ionizing radiation is a known risk factor for secondary sarcomas that localize to bone and can present as a nontender anterior sternal mass. Radiation-induced osteosarcomas are usually high grade and confer a poorer prognosis, especially when axially located. Cinematic rendering is a unique resource for radiologists and surgeons to improve evaluation and surgical planning for radiation-induced osteosarcomas.

## Patient consent

The patient reported in the manuscript signed the informed consent/authorization for participation in research, which includes the permission to use data collected in future research projects including presented case details and images used in this manuscript.
